# Construction of a high-density genetic map for faba bean (*Vicia faba* L.) and quantitative trait loci mapping of seed-related traits

**DOI:** 10.3389/fpls.2023.1201103

**Published:** 2023-06-07

**Authors:** Na Zhao, Dong Xue, Yamei Miao, Yongqiang Wang, Enqiang Zhou, Yao Zhou, Mengnan Yao, Chunyan Gu, Kaihua Wang, Bo Li, Libin Wei, Xuejun Wang

**Affiliations:** Department of Economic Crops, Jiangsu Yanjiang Institute of Agricultural Science, Nantong, China

**Keywords:** *vicia faba* L., single nucleotide polymorphisms (SNP), high-density genetic map, seed related traits, quantitative trait loci (QTL)

## Abstract

Faba bean (*Vicia faba* L.) is a valuable legume crop and data on its seed-related traits is required for yield and quality improvements. However, basic research on faba bean is lagging compared to that of other major crops. In this study, an F_2_ faba bean population, including 121 plants derived from the cross WY7×TCX7, was genotyped using the Faba_bean_130 K targeted next-generation sequencing genotyping platform. The data were used to construct the first ultra-dense faba bean genetic map consisting of 12,023 single nucleotide polymorphisms markers covering 1,182.65 cM with an average distance of 0.098 cM. The map consisted of 6 linkage groups, which is consistent with the 6 faba bean chromosome pairs. A total of 65 quantitative trait loci (QTL) for seed-related traits were identified (3 for 100-seed weight, 28 for seed shape, 12 for seed coat color, and 22 for nutritional quality). Furthermore, 333 candidate genes that are likely to participate in the regulation of seed-related traits were also identified. Our research findings can provide a basis for future faba bean marker-assisted breeding and be helpful to further modify and improve the reference genome.

## 1 Introduction

Faba bean (*Vicia faba* L.), also called horse bean, is a member of the Fabaceae family (grain legume) that originated in the Near East, and is an important cool-season food legume (Cubero, 1974). It is currently widely cultivated in Africa, Asia, Europe, Australia, and North America (Alghamdi et al., 2012). Faba bean can be used as a green manure as it has nitrogen fixation capabilities and can thus improve soil quality (Jensen et al., 2010). Additionally, faba bean is used as a type of food for humans and as a feed for animals (Martineau-Côté et al., 2022), and the fresh seeds can be consumed as vegetables (Zong et al., 2009). Furthermore, due to its rich nutritional value and high protein and lysine content, it can be effectively utilized as a source of plant protein (Etemadia et al., 2019), and is also rich in phenols (Amarowicz and Shahidi, 2017). The edible part of the seed thus directly affects its yield and quality. It is therefore important to clarify the genetic basis of the related traits in faba bean breeding programs. The phenotypic and quality traits of faba bean seeds are mostly complex and easily affected by the environment, and consequently, the use of molecular technologies is required to fully understand them. The genetic linkage map is an effective tool that can help to improve our understanding of the inheritance of traits at a genome-wide level (Verma et al., 2015). Furthermore, the fine mapping of quantitative trait loci (QTL) and candidate genes related to specific traits has traditionally been performed using high-resolution genetic linkage maps (Zhang et al., 2016).

Faba bean has one of the largest genomes among crop legumes, and is diploid with 2n = 12 chromosomes and a large genome of 13,000 Mb (Johnston et al., 1999). As a result, basic research on faba bean is lagging behind that of other major crops that have relatively complete genetic maps, such as maize (*Zea mays* L.), rice (*Oryza sativa* L.), and wheat (*Triticum aestivum* L.) (Wang H et al., 20122). Initially, some traditional markers, including morphological and isoenzyme, random amplified polymorphic DNA, and microsatellite markers, were used to construct several faba bean genetic maps (Torres et al., 1993; Satovic et al., 1996; Patto et al., 1999; Román et al., 2002; Ávila et al., 2004; Román et al., 2004; Ávila et al., 2005; Ellwood et al., 2008; Díaz-Ruiz et al., 2009; Díaz-Ruiz et al., 2010; Cruz-Izquierdo et al., 2012; Gutiérrez et al., 2013). With the development of high-throughput sequencing technologies, simple sequence repeats (SSR) and single nucleotide polymorphisms (SNP) have been extensively used to construct genetic maps and identify QTLs in faba beans (Arbaoui et al., 2008; Ma et al., 2013; Satovic et al., 2013; Kaur et al., 2014; Sallam et al., 2016; Webb et al., 2016; Catt et al., 2017; Ocaña−Moral et al., 2017; Yang et al., 2019). Sudheesh et al. (2019) constructed an integrated genetic map for faba bean spanning 1,439 cM, with an average distance of 0.80 cM per marker using a total of 1,850 markers. Carrillo-perdomo et al. (2020) constructed a high-density genetic map containing gene-based SNP markers with a length of 1,547.71 cM, and an average distance of 0.89 cM. Recently, an integrated genetic linkage map containing 6,895 SNPs, with a length of 3,324.48 cM was constructed from two F_2_ populations by Li et al. (2023).The construction of a fine linkage map for faba bean can greatly improve the efficiency of related genetic research and crop breeding and enable the establishment of marker selection and QTL mapping associated with economically important traits (Khazaei et al., 2014; Aguilar−Benitez et al., 2021; Gutierrez and Torres, 2021; Carrillo-Perdomo et al., 2022).

To date, although there have been some studies on QTL mapping associated with seed-related traits in faba bean, few related genes have been mapped. The QTL associated with 100-seed weight was first identified on chromosome 6 and significantly correlated with 20 markers (Patto et al., 1999). Furthermore, Ávila et al. (2017) identified five QTLs related to 100-seed weight. The F_2_ populations generated from Yun122 and TF42 were used to construct genetic maps, and four QTLs controlling seed length, width, and 100-seed weight were identified (Tian et al., 2018). Macas et al. 1993a mapped the chromosomal positions of genes encoding seed storage proteins. Gutierrez et al. (2007) identified two SCAR markers tightly linked to a gene controlling tannin deficiency in faba beans and Hou et al. (2018) screened one SSR marker (SSR84) closely linked to the tannin content (zt-1) gene using 596 SSR markers and 100 ISSR markers, which could aid in accurate prediction of the zt-1 genotypes. Recently, 15 markers were identified with seed size associations based on genome-wide association study (Jayakodi et al., 2023). Li et al. (2023) identified 32 QTLs related to seed size and 6 QTLs related to seed coat color.

The efficiency and precision of QTL mapping are restricted by the low density of molecular markers in the resulting genetic maps; however, this can be addressed using high-throughput DNA microarray (DNA chip) technologies. Wang et al. (2021) utilized a large-scale transcriptome and a large number of SNP markers to develop the Faba_bean_ 130 K SNP targeted next-generation sequencing (TNGS) genotyping platform, which contains 130,514 SNPs and can be used for high-density genetic linkage map development and QTL mapping.

In this study, an ultra-dense genetic map from an F_2_ population was constructed using the Faba_bean_ 130 K SNP TNGS genotyping platform. QTLs for 15 seed-related traits, including 100-seed weight (HSW), seed area (SA), seed perimeter (SP), seed length (SL), seed width (SW), seed length and width ratio (SLWR), seed thickness (ST), seed coat color R value (SC-R), seed coat color G value (SC-G), seed coat color B value (SC-B), protein content (PC), starch content (StC), fiber content (FC), lipid content (LC), and tannin content (TC), which were mapped based on the phenotypic data from F_2_ and F_2:3_ populations. The ultra-dense genetic map and QTLs produced from this study can be used for faba bean marker-assisted selection (MAS), gene mapping, and reference genome improving. MAS is a method used in plant breeding, once the linkage has been established between physical markers and the target traits, individuals with desirable traits can be selected by detecting the molecular markers.

## 2 Article types

This article was submitted to Plant Breeding, a section of the journal Frontiers in Plant Science.

## 3 Materials and methods

### 3.1 Plant materials and phenotypic data evaluation

An interspecific F_2_ population containing 121 individual plants was generated from WY7 and TCX7 parent materials. The female parent WY7 is a germplasm resource introduced from the UK with a medium seed size and dark-purple seed coat color. The male parent TCX7 has a large seed size, with a white seed coat, is of good quality, and is cultivated by the Jiangsu Yanjiang Institute of Agricultural Sciences, China. The F_2_ individual plants and their parents were planted in Xueyao, Jiangsu Province, China from 2020–2021, and the F_2:3_ plant lines and their parents were planted in Xueyao and Jiuhua respectively, Jiangsu Province, China, from 2021–2022. Each faba bean line was planted in one row of 2.4 m in length, with a row distance of 0.8 m, and plant spacing of 0.2 m. Field management was consistent with local production practices throughout the whole growth period. Ten seed phenotypic traits and five nutritional quality traits of the parents, F_2_ individual plants, and F_2:3_ families in two environments (Xueyao and Jiuhua) were investigated. Ten plants in each F_2:3_ line and their parents were harvested. The seed shape traits assessed were SA, SP, SL, SW, SLWR, and ST. The average indicators of HSW, SA, SP, SL, SW, and SLWR used an automatic seed testing system (SC-A1, Hangzhou Wanshen Detection Technology Co., Ltd., Hangzhou, China). The average values of the 10 thickest parts of the seeds were regarded as ST. The seed coat color traits including SC-R, SC-G, and SC-B were measured by spectrophotometer (YS3020, 3NH, China). Nutritional quality traits PC, StC, FC, LC, and TC were determined using a DA7250 NIR analyzer (Perten Instruments, Hägersten, Sweden) with three replicates.

Statistical analysis of the data, such as frequency distribution, coefficient of variation, standard deviation, skewness and kurtosis analysis, was performed using the ANOVA function of IciMapping 4.2.53. The phenotypic correlation between these traits was obtained by Pearon’s correlation analyses using SPSS software. Ver. 26 (IBM SPSS Statistics, Chicago, IL, USA) and R software (version 3.2.2, http://www.r-project.org).

### 3.2 Genotyping

The total genomic DNA of the F_2_ individuals and their parental lines was extracted from fresh leaves using the CTAB method (Doyle and Doyle, 1987). A NanoDrop spectrophotometer (Thermo Fisher Scientific, USA) was used to determine the optical density ratios of OD260/280 (>1.8) and OD260/230 >1.5). A Qubit was used for precise quantification, and gel electrophoresis was used to monitor and assess the quality and contamination of all DNA samples. The Illumina sequencing library was constructed by binding biotin-labeled RNA probes to spliced DNA fragments using restriction enzymes and was sequenced using the China Golden Maker (Beijing) Biotech Co. Clean data were derived from the raw sequencing data after quality control (filter parameters: trimmomatic-0.36.jar PE -phred33 ILLUMINACLIP: fa: 2:30:10:8: true LEADING:3 TRAILING:3 SLIDINGWINDOW: 4:15 MINLEN:100) and then matched to the faba bean transcriptome (Wang et al., 2021) by using BWA software (version 0.7.17) with parameters: MEM -T 4 -K 32 -M -R). Based on the results of the sequence alignment, SNPs from the populations genomic data were detected with GATK (version 4.1.2.0) and filtered with VCFtools (version 0.1.13). The detailed criteria and analysis methods were in accordance with Wang et al. (2021).

### 3.3 Construction of the genetic map

The harvested genotypes of the samples were firstly filtered before genetic map construction. Based on the filtered genotypes, for each loci, the individuals were coded as “A” (if same with parent TCX7), “B” (same with the parent WY7), “H” (heterozygous containing 2 alleles from each of the parents) or “missing” (all other scenarios). The discarded loci include 1) the loci which were heterozygous in either parent, and 2) the loci with the missing rate above 80% in the population. This was done by using a python script from Li et al. (2021). The genetic map construction used a similar procedure as Li et al. (2021). Briefly, the coded “ABH” genotype matrix was firstly filtered to discarding distortion loci with the threshold P value = 0.01, and then was fed to Lep-Map3 (Rastas, 2017). The default parameters and a logarithm of odds (LOD) score of 12 were used in Lep-Map3. Linkage groups (LGs) with markers less than 100 were removed, and Kosambi function was applied to covert the recombinant rate into LG length (cM, centi-Morgan).

### 3.4 QTL mapping

Based on the genetic map constructed above and the phenotypes from multiple environments, we conducted QTL mapping in 2 programs, i.e. QTL Cartographer 2.5 (Wang S et al., 2012) and IciMapping 4.2.53 (Meng et al., 2015). In QTL Cartographer, CIM (Composite interval mapping) method was used, and the parameters were set up as: control markers = 5, window size = 10.0 cM, walk speed = 1.0 cM, and the LOD threshold was determined by 500 times permutation tests. For the Icimapping program, ICIM (Inclusive composite interval mapping) method was selected, and the flowing parameters were used: “missing phenotype = Deletion”, “mapping step = 1 cM” and “LOD threshold = 1000 times permutation at type I error 0.05”. In the mapping result, VG/VP value reflects the explanation rate of phenotypic variance, and the confidence interval of a QTL was determined by the outermost 2 markers above threshold. The QTLs were named as follows: q + trait abbreviation + chromosome number + QTL number.

### 3.5 Candidate gene identification and annotation

The splice junction sequences in the Faba_bean_ 130 K SNP TNGS genotyping platform were searched within the QTL intervals and then mapped to 243,120 unigenes (Wang et al., 2021), which were referred to in order to obtain the candidate genes and their gene annotations.

### 3.6 Reference genome mapping

Sequence of the genes in genetic map alignment with reference genome (https://projects.au.dk/fabagenome/genomics-data) and the candidate genes were visual mapped to the reference genome using TB tools software.

## 4 Results

### 4.1 Phenotypic analyses

**Figure 1:**
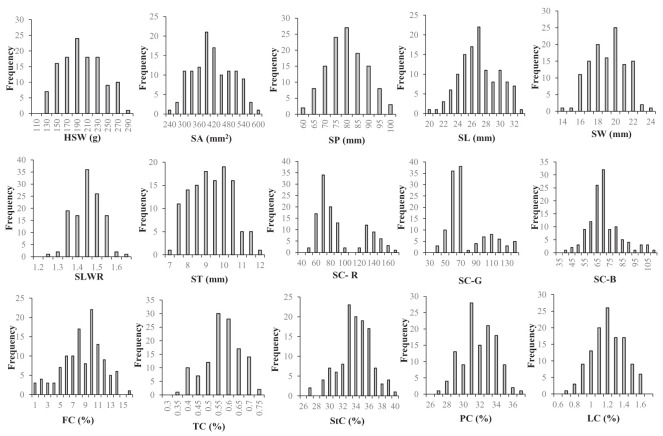
Frequency distributions of seed-related traits in 121 F_2_ derived from a cross between WY7 andTCX7. HSW 100-seed weight (g), SA seed surface area (mm^2^), SP seed perimeter (mm), SL seed length (mm), SW seed width (mm), SLWR seed length and width ratio, ST seed thickness (mm), SC-R seed coat color R value, SC-G seed coat color G value, SC-B seed coat color B value, FC fiber content (%), TC tannin content (%), StC starch content (%), PC protein content (%), LC lipid content (%).

The two parent materials showed significant differences in HSW, SA, SP, SL, SW, SC-R, SC-G, SC-B, FC, TC, StC and LC (**Table 1**). The statistical results of the phenotypic variations in the seed-related traits among the parents, F_2_ populations, and F_2:3_ individuals (**Supplementary Table S1**) suggested that HSW, SA, SP, SL, SW, SLWR, PC, StC, FC, LC, and TC showed continuous variation. The absolute values of skewness and kurtosis were almost less than 1, approximately conforming to the normal distribution, meeting the requirements of QTL analysis (**Figure 1**; **Table 1**).

**Table 1:** Details of average of F_2_, two environments of F_2:3_ individuals and their parents.

Trait	Parents		Population							
	WY7	TCX7	Min	Max	Mean	SD	Variance	CV%	Skewness	Kurtosis
**HSW**	133.05**	245.54**	113.62	230.34	170.20	27.72	762.07	16.29	0.23	-0.69
**SA**	290.20 **	526.83 **	245.00	484.25	360.25	59.28	3,485.39	16.46	0.24	-0.85
**SP**	65.46**	89.15 **	55.94	87.59	73.38	6.53	42.26	8.90	0.08	-0.59
**SL**	22.56 **	30.61 **	19.18	29.60	25.22	2.13	4.52	8.46	0.00	-0.46
**SW**	16.00**	21.62**	13.56	20.88	17.63	1.55	2.37	8.77	0.09	-0.65
**SLWR**	1.43	1.42	1.30	1.53	1.44	0.05	0.00	3.46	-0.33	-0.35
**ST**	9.03	9.97	6.67	10.92	8.93	0.71	0.49	7.91	-0.03	0.28
**SC-R**	64.22**	136.62 **	53.34	158.93	94.16	31.80	1,002.93	33.77	0.64	-1.12
**SC-G**	56.89*	119.36 *	46.84	137.25	80.35	25.73	656.80	32.03	0.70	-0.98
**SC-B**	61.94 *	91.37 *	54.26	104.06	74.90	11.92	141.00	15.92	0.49	-0.70
**FC**	5.67 *	10.95*	3.02	12.83	7.98	2.06	4.19	25.78	-0.15	-0.35
**TC**	0.53 **	0.65 **	0.43	0.72	0.57	0.06	0.00	9.84	0.16	-0.27
**StC**	32.66 *	35.95 *	29.56	37.58	33.95	1.53	2.32	4.50	-0.23	0.42
**PC**	30.90	31.40	28.19	34.05	31.12	1.22	1.48	3.93	-0.19	-0.29
**LC**	1.30 *	1.01 *	0.83	1.48	1.12	0.13	0.02	11.53	0.07	-0.23

SD standard deviation, CV coefficient of variation, HSW 100-seed weight (g), SA seed surface area (mm^2^), SP seed perimeter (mm), SL seed length (mm), SW seed width (mm), SLWR seed length and width ratio, ST seed thickness (mm), SC-R seed coat color R value, SC-G seed coat color G value, SC-B seed coat color B value, FC fiber content (%), TC tannin content (%), StC starch content (%), PC protein content (%), LC lipid content (%). Significant differences between two parental lines WY7 and TCX7 are marked by * and **, which were determined by the Student’s t test at P < 0.05 and P < 0.01, respectively.

### 4.2 Correlation analyses among different traits

**Figure 2:**
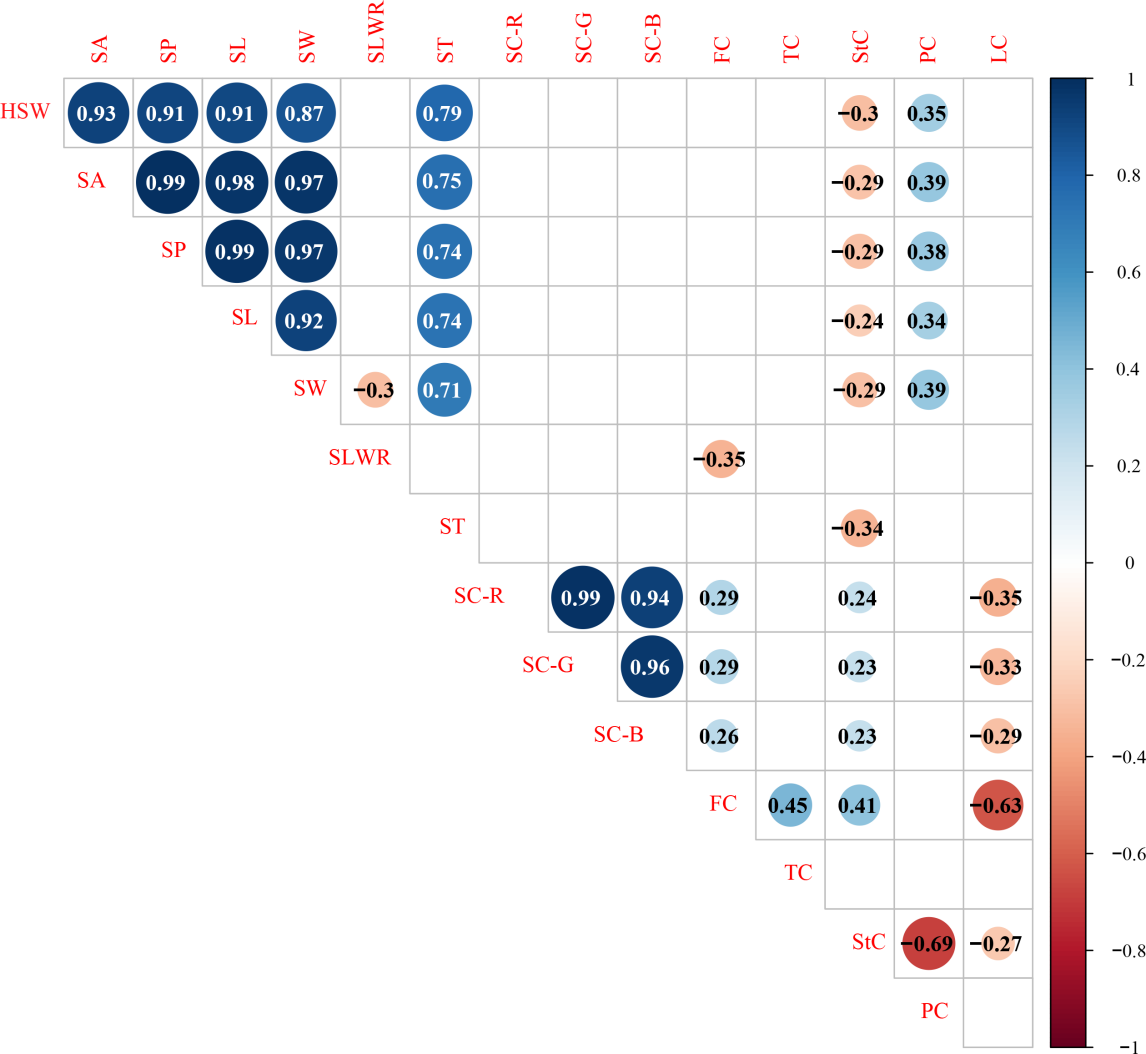
Correlation analysis of different traits in 0.01 probability level.HSW 100-seed weight (g), SA seed surface area (mm^2^), SP seed perimeter (mm), SL seed length (mm), SW seed width (mm), SLWR seed length and width ratio, ST seed thickness (mm), SC-R seed coat color R value, SC-G seed coat color G value, SC-B seed coat color B value, FC fiber content (%), TC tannin content (%), StC starch content (%), PC protein content (%), LC lipid content (%).

Significant Pearson’s correlations (p < 0.01) for the same trait showed a significant positive relationship between the F_2_ and F_2:3_ populations in Xueyao and Jiuhua (**Supplementary Table S2**). Phenotypic correlations (p < 0.01) among the different traits are shown in **Figure 2**. Seed shape traits, including HSW, SA, SP, SL, and SW, were positively correlated with PC, and negatively correlated with StC. Seed coat color traits, including SC-R, SC-G, and SC-B, were positively correlated with FC and StC and negatively correlated with LC. There was no significant correlation between the seed shape traits and seed coat color traits in this study.

### 4.3 Genetic map construction

**Figure 3:**
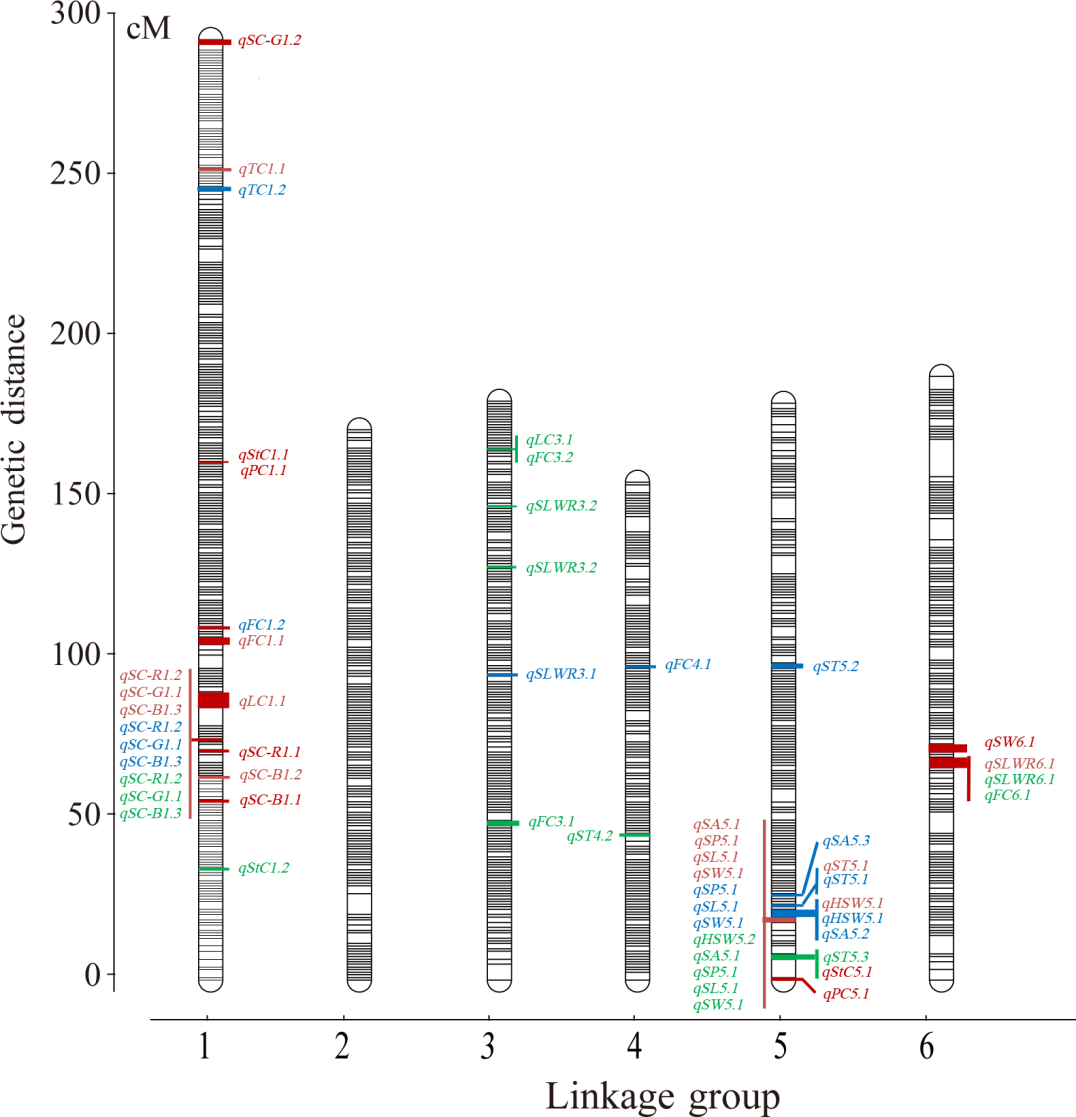
The ultra−high density genetic linkage map of faba bean based on bin makers and QTLs of seed-related traits. Note: Only the QTLs with the phenotypic variation > 10% were shown. Red words means QTLs detected in the F_2_, blue words means QTLs detected in the F_2:3_ of Jiuhua and green words means QTLs detected in the F_2:3_ of Xueyao.

A total of 121 F_2_ plants and their parents were genotyped using 130,514 SNPs in the Faba_bean_ 130 K SNP TNGS genotyping platform, showing excellent results, quality, and matching scores (**Supplementary Tables S3**; **S4**). There were 12,023 SNP-tagged gene microarrays with polymorphism between parents (**Supplementary Table S5**), and they were successfully genotyped into “A,” “B,” and “H” types in the population. All co-isolated markers were defined as one bin, and 1106 bin markers were used to construct a genetic map containing 6 LGs. The overall length of the genetic map was 1,182.65 cM with an average marker spacing of 0.098 cM. Each LG range was from 157.08–296.82 cM, and the average distance between markers was from 0.079–0.114 cM. LG1 had the largest number of markers with 3,325 SNPs. The smallest gap identified in the map was 0.826 cM, the total number of gaps > 5 cM was 9, and the largest gap was 11.78 cM LG6. Additionally, the ratio of marker intervals < 5 cM for all LGs was > 97% (**Figure 3**; **Table 2**).

**Table 2:** Summary of the consensus reference genetic map of faba bean in this study.

Linkage groups	SNP count	Bin count	Length (cM)	Average interval (cM)	Largest gap size (cM)	Numbers of gaps > 5 cM
**LG1**	3,325	285	296.818	0.089	5.811	1
**LG2**	2,207	173	173.630	0.079	6.651	1
**LG3**	2,121	184	182.697	0.086	4.975	0
**LG4**	1,758	157	157.084	0.089	4.975	0
**LG5**	1,597	156	181.963	0.114	6.650	3
**LG6**	1,015	151	190.461	0.188	11.784	4
**Total**	12,023	1106	1182.653	0.098	11.784	9

### 4.4 QTL analysis

QTL mapping was performed using QTL lciMapping and QTL-Cart CIM, and 65 (**Supplementary Table S6**) and 50 (**Supplementary Table S7**) QTLs were identified for all 15 seed-related traits detected in the F_2_ and F_2:3_ populations, respectively. Together, these two mapping strategies identified 28 overlapping QTLs (**Supplementary Table S8**). Of these, the QTL intervals observed using the CIM method were usually wider, whereas the intervals from the ICIM method were narrower. Consequently, the results obtained using the lCIM method were used in this study. The genetic effect (the explanation rate of phenotyte variance or VG/VP) of the QTLs detected using ICIM for 15 seed-related traits ranged from 4.90–73.99%, with peak LOD values ranging from 4.48–35.25 (**Supplementary Table S6**). Among the 65 loci, there were 11 QTLs that were detected for more than two traits (**Supplementary Table S9**). There were 39 QTLs identified that individually accounted for > 10% of the phenotypic variation (**Table 3**) and 1 QTL explained < 5% of the phenotypic variation (**Supplementary Table S6**). A total of 41 and 21 QTLs were found to have positive and negative additive effects, respectively.

**Table 3:** QTLs distribution of 15 seed-related traits with responsible for more than 10% of the explained phenotypic variation.

Trait	LG	QTL	environment	Left maker		Right maker		VG/VP (%)	Peak LOD	Add
				name	pos	name	pos			
**HSW**	5	*qHSW5.1*	F_2_(2020)	yDN135233_c2_g1_2027	19.5	yDN120969_c0_g1_320	22.5	35.84	17.81	345.255
			F_2:3_-JH(2021)	yDN135233_c2_g1_2027	19.5	yDN120969_c0_g1_320	22.5	38.88	15.79	214.079
		*qHSW5.2*	F_2:3_-XY(2021)	yDN151173_c1_g2_816	18.5	yDN135233_c2_g1_2027	19.5	25.71	10.42	228.937
**SA**	5	*qSA5.1*	F_2_(2020)	yDN151173_c1_g2_816	18.5	yDN135233_c2_g1_2027	19.5	51.51	18.41	81.633
			F_2:3_-XY(2021)	yDN151173_c1_g2_816	18.5	yDN135233_c2_g1_2027	19.5	40.33	15.13	54.410
		*qSA5.2*	F_2:3_-JH(2021)	yDN135233_c2_g1_2027	18.5	yDN120969_c0_g1_320	22.5	39.64	24.81	49.392
		*qSA5.3*	F_2:3_-JH(2021)	hDN150254_c0_g5_96	26.5	dDN52935_c3_g4_215	28.5	15.19	11.34	5.348
**SP**	5	*qSP5.1*	F_2_(2020)	yDN151173_c1_g2_816	18.5	yDN135233_c2_g1_2027	19.5	46.06	15.55	8.962
			F_2:3_-JH(2021)	yDN151173_c1_g2_816	18.5	yDN135233_c2_g1_2027	19.5	55.47	23.55	5.636
			F_2:3_-XY(2021)	yDN151173_c1_g2_816	18.5	yDN135233_c2_g1_2027	19.5	40.13	14.82	5.828
**SL**	5	*qSL5.1*	F_2_(2020)	yDN151173_c1_g2_816	18.5	yDN135233_c2_g1_2027	19.5	42.65	14.06	2.724
			F_2:3_-JH(2021)	yDN151173_c1_g2_816	18.5	yDN135233_c2_g1_2027	19.5	48.32	28.30	1.756
			F_2:3_-XY(2021)	yDN151173_c1_g2_816	18.5	yDN135233_c2_g1_2027	19.5	35.96	13.79	1.904
**SW**	5	*qSW5.1*	F_2_(2020)	yDN151173_c1_g2_816	18.5	yDN135233_c2_g1_2027	19.5	39.37	14.92	1.857
			F_2:3_-JH(2021)	yDN151173_c1_g2_816	18.5	yDN135233_c2_g1_2027	19.5	42.93	22.55	1.165
			F_2:3_-XY(2021)	yDN151173_c1_g2_816	18.5	yDN135233_c2_g1_2027	19.5	36.78	12.90	1.356
	6	*qSW 6.1*	F_2_(2020)	yDN128644_c0_g1_417	71.5	hDN154119_c0_g2_776	75.5	10.69	5.07	0.866
**SLWR**	3	*qSLWR 3.1*	F_2:3_-JH(2021)	yDN154982_c0_g1_462	95.5	yDN150491_c1_g1_2729	96.5	18.23	6.37	0.004
		*qSLWR 3.2*	F_2:3_-XY(2021)	hDN149791_c1_g1_342	129.5	yDN133005_c0_g1_512	130.5	17.24	11.11	-0.036
		*qSLWR 3.3*	F_2:3_-XY(2021)	yDN155504_c1_g2_308	147.5	yDN155504_c1_g2_253	149.5	16.78	9.38	-0.036
	6	*qSLWR 6.1*	F_2_(2020)	yDN145987_c0_g1_369	67.5	yDN138086_c0_g1_82	71.5	25.02	7.34	-0.046
			F_2:3_-XY(2021)	yDN145987_c0_g1_369	67.5	yDN138086_c0_g1_82	70.5	10.40	6.21	0.032
**ST**	4	*qST4.1*	F_2:3_-XY(2021)	hDN131761_c0_g1_1016	45.5	hDN122802_c0_g1_447	46.5	16.90	12.25	0.127
	5	*qST5.1*	F_2_(2020)	yDN131562_c0_g2_1383	23.5	dDN54339_c2_g2_203	24.5	24.78	7.29	0.646
			F_2:3_-JH(2021)	yDN131562_c0_g2_1383	23.5	dDN54339_c2_g2_203	24.5	19.19	9.38	0.452
		*qST5.2*	F_2:3_-JH(2021)	hDN154491_c1_g8_222	98.5	hDN152331_c2_g3_311	99.5	10.07	4.78	0.123
		*qST5.3*	F_2:3_-XY(2021)	hDN148575_c1_g1_723	7.5	yDN151173_c1_g2_935	9.5	10.66	8.58	0.475
**SC-R**	1	*qSC-R1.1*	F_2_(2020)	hDN132853_c1_g2_224	71.5	dDN45140_c0_g1_2482	72.5	68.60	35.13	24.007
		*qSC-R1.2*	F_2_(2020)	hDN125239_c1_g4_1476	75.5	yDN127251_c0_g1_756	76.5	12.90	11.02	11.943
			F_2:3_-JH(2021)	hDN125239_c1_g4_1476	75.5	yDN127251_c0_g1_756	76.5	73.99	34.76	35.411
			F_2:3_-XY(2021)	hDN125239_c1_g4_1476	75.5	yDN127251_c0_g1_756	76.5	65.35	29.45	34.151
**SC-G**	1	*qSC-G1.1*	F_2_(2020)	hDN125239_c1_g4_1476	75.5	yDN127251_c0_g1_756	76.5	51.48	35.25	17.308
			F_2:3_-JH(2021)	hDN125239_c1_g4_1476	75.5	yDN127251_c0_g1_756	76.5	66.97	28.88	25.116
			F_2:3_-XY(2021)	hDN125239_c1_g4_1476	75.5	yDN127251_c0_g1_756	76.5	68.34	33.01	36.541
		*qSC-G1.2*	F_2_(2020)	yDN157063_c3_g3_806	295.5	yDN157063_c3_g3_836	296	12.36	12.19	-11.891
**SC-B**	1	*qSC-B1.1*	F_2_(2020)	yDN147029_c0_g1_601	56.5	yDN142452_c3_g4_331	57.5	24.62	15.73	9.483
		*qSC-B1.2*	F_2_(2020)	hDN135643_c3_g1_514	63.5	yDN151467_c2_g1_440	64.5	22.55	16.11	7.153
		*qSC-B1.3*	F_2_(2020)	hDN125239_c1_g4_1476	75.5	yDN127251_c0_g1_756	76.5	10.63	8.93	-6.273
			F_2:3_-JH(2021)	hDN125239_c1_g4_1476	75.5	yDN127251_c0_g1_756	76.5	37.04	12.09	9.231
			F_2:3_-XY(2021)	hDN125239_c1_g4_1476	75.5	yDN127251_c0_g1_756	76.5	54.25	20.22	16.479
**FC**	1	*qFC1.1*	F_2_(2020)	yDN145946_c2_g3_400	107.5	yDN125063_c0_g1_87	108.5	21.37	9.44	2.049
		*qFC1.2*	F_2:3_-JH(2021)	dDN53089_c3_g1_46	109.5	yDN129665_c0_g3_238	110.5	14.85	6.26	1.379
	3	*qFC3.1*	F_2:3_-XY(2021)	yDN148417_c0_g1_302	48.5	hDN142257_c0_g1_1594	49.5	15.43	5.97	1.057
		*qFC3.2*	F_2:3_-XY(2021)	yDN119514_c0_g1_312	166.5	hDN145176_c0_g1_497	167.5	13.65	5.32	-0.962
	4	*qFC4.1*	F_2:3_-JH(2021)	hDN122239_c0_g2_1049	98.5	hDN154311_c1_g1_631	99.5	10.16	4.97	1.008
	6	*qFC6.1*	F_2:3_-XY(2021)	yDN145987_c0_g1_369	67.5	yDN138086_c0_g1_82	71.5	10.82	4.65	0.686
**TC**	1	*qTC1.1*	F_2_(2020)	hDN124375_c0_g1_451	255.5	dDN47789_c0_g1_281	256.5	22.70	6.46	-0.061
		*qTC1.2*	F_2:3_-JH(2021)	hDN122621_c4_g3_51	249.5	yDN154539_c0_g2_558	251.5	17.61	5.17	-0.034
**StC**	1	*qStC1.1*	F_2_(2020)	dDN40232_c0_g1_396	162.5	yDN134012_c0_g1_800	163.5	13.77	8.19	-1.306
		*qStC1.2*	F_2:3_-XY(2021)	hDN148143_c3_g2_213	34.5	yDN141447_c5_g1_240	35.5	18.44	5.53	1.041
	5	*qStC5.1*	F_2_(2020)	hDN148575_c1_g1_723	7.5	yDN151173_c1_g2_935	9.5	11.38	6.64	-1.192
**PC**	1	*qPC1.1*	F_2_(2020)	dDN40232_c0_g1_396	162.5	yDN134012_c0_g1_800	163.5	17.32	6.12	1.070
	5	*qPC5.1*	F_2_(2020)	dDN41265_c0_g1_1106	0	dDN41265_c0_g1_1104	0.5	13.65	4.99	0.875
**LC**	1	*qLC1.1*	F_2_(2020)	hDN155223_c0_g1_2012	86.5	hDN146106_c2_g1_2134	88.5	20.78	5.91	-0.141
	3	*qLC3.1*	F_2:3_-XY(2021)	yDN119514_c0_g1_312	166.5	hDN145176_c0_g1_497	167.5	21.35	12.72	-0.130

The QTL with underlines means stable QTL for each trait. HSW 100-seed weight (g), SA seed surface area (mm^2^), SP seed perimeter (mm), SL seed length (mm), SW seed width (mm), SLWR seed length and width ratio, ST seed thickness (mm), SC-R seed coat color R value, SC-G seed coat color G value, SC-B seed coat color B value, FC fiber content (%), TC tannin content (%), StC starch content (%), PC protein content (%), LC lipid content (%), JH Jiuhua, XY Xueyao.

#### 4.4.1 Seed morphology traits

Three QTLs of HSW were detected and had peak LOD scores of 4.64–17.81, which explained 7.26%–38.88% of the HSW variation. One was located on LG4, and two were mapped to LG5 (**Table 3**). QTLs detected more than two times among F_2_, F_3_-XY and F_3_-JH were considered environmentally stable. *qHSW5.1* was detected in F_2_ and F_3_-JH (**Table 3**; **Supplementary Table S10**).

A total of 28 QTLs were found for several seed shape traits, and 6 were regarded as stable (**Table 3**; **Supplementary Table S10**). Five QTLs were detected on LG5 with a peak LOD score of 4.69–24.81, and they explained 4.90–51.51% of the SA variation. *qSA5.1* was detected in F_2_ and F_3_-XY. Only one environmentally stable QTL (*qSP5.1*) of SP was identified on LG5 with a peak LOD score ranging from 14.82–23.55, and it explained 40.13–55.47% of the SP variation. Four QTLs associated with SL had peak LOD scores ranging 4.73–28.30, which explained 5.50–48.32% of the SL variation and were located on LG1, LG3, LG5, and LG6. According to the results, *qSL5.1* was a stable QTL, which detected in F_2_, F_3_-XY and F_3_-JH.

Five QTLs explained 6.64–42.93% of the SW variance, with peak LOD scores ranging from 4.48–22.55, which were identified in linkage groups LG2 (1), LG3 (1), LG5 (1), and LG6 (2). An environmentally stable QTL (*qSW5.1*) was also identified. Five QTLs explained 9.59–25.02% of the SLWR variance, and the peak LOD scores varied from 5.33–11.11, and *qSW6.1* was stable.

For ST, eight QTLs were detected in LG4 (3), LG5 (3), and LG6 (2), with LOD scores ranging from 4.48–12.25, and they explained 6.65–24.78% of the total phenotypic variation. *qST5.1* was detected in F_2_, F_3_-XY and F_3_-JH. Among these QTLs, four were overlapping for seed shape traits.

For seed coat color traits, 12 QTLs were detected, including 3, 5, and 4 QTLs for R, G, and B, respectively. The phenotypic variation explained by each individual QTL ranged from 5.00–73.99%, with a peak LOD of 4.53–35.25 (**Table 3**). Three were overlapping QTLs and one was a stable QTL, both located in linkage group LG1 (**Supplementary Table S10**).

#### 4.4.2 Nutritional quality traits

The results from the QTL analysis identified 22 QTLs associated with nutritional quality traits (**Table 3**; **Supplementary Table S10**), 7 QTLs explained 9.25–21.35% of the FC variance, 2 QTLs explained 22.70–17.61% of the TC variance, 7 QTLs explained 7.09–18.44% of the StC variance, 2 QTLs explained 13.65–17.32% of the PC variance, and 4 QTLs explained 6.74–21.35% of LC variance. *qFC3. 3* was considered stable.

### 4.5 Analysis of candidate genes

The genes in the QTL intervals were screened using the Faba_bean_ 130 K SNP TNGS genotyping platform (**Table 4**). The results showed that 333 genes and 610 SNPs were detected at 65 QTL intervals. Among the 333 genes, HSW, seed shape, seed coat color, and nutritional quality traits contained 8, 117, 100, and 109 genes, respectively, and 173 genes were functionally annotated by database comparison. A total of 67 candidate genes within the environmentally stable QTL intervals were detected, including 2, 20, 39, 3 genes related to HSW, seed shape, seed coat color, and nutritional quality traits, respectively. The results showed that 213 genes in 41 QTLs explained > 10% of the observed phenotypic variance, and they were further assessed (**Supplementary Table S11**). There were 6 genes related to HSW within these QTL intervals, and 5 were annotated, including the CCCH-type zinc finger protein and calcium-binding protein. There were 53 seed shape-related genes and 30 genes were annotated, including serine/threonine phosphatase, bHLH transcription factor, calcium-binding protein Ca^2+^/H^+^-exchanging protein, and other functional genes. Seed color-related genes included 39 and 19 genes that were annotated, including ubiquitin-like protein, the WD40 family, and transcription factors. There were 79 genes associated with nutritional quality traits, and 41 genes were annotated, including numerous genes encoding enzymes, functional genes, and some transcription factors.

**Table 4:** Details of genes and SNPs of 15 seed-related traits in QTL interval based on 130K TNGS.

Trait	Total QTL number	SNP number	Gene number	VG/VP >10 QTLs interval Gene number	Stable QTLs interval gene number
**HSW**	3	15	8	6	5
**SA**	5	23	13	9	2
**SP**	1	4	2	2	2
**SL**	4	37	20	2	2
**SW**	5	31	22	9	2
**SLWR**	5	38	20	15	2
**ST**	8	61	40	23	10
**SC-R**	3	74	30	25	13
**SC-G**	5	88	42	14	13
**SC-B**	4	63	28	21	13
**FC**	7	28	20	17	3
**TC**	2	91	49	49	0
**StC**	7	30	21	10	0
**PC**	2	5	4	4	0
**LC**	4	22	15	7	0
**Total**	65	610	333	213	67

HSW 100-seed weight (g), SA seed surface area (mm^2^), SP seed perimeter (mm), SL seed length (mm), SW seed width (mm), SLWR seed length and width ratio, ST seed thickness (mm), SC-R seed coat color R value, SC-G seed coat color G value, SC-B seed coat color B value, FC fiber content (%), TC tannin content (%), StC starch content (%), PC protein content (%), LC lipid content (%).

### 4.6 Reference genome mapping

Sequences of gene in our genetic map were well alignment with the recent published reference genome of faba bean (**Supplementary Table S12**). It was found that about 60% of the genes in each LG were mapped to the corresponding chromosome. Specifically, LG1–LG6 were assigned to chromosome 1, chromosome 3, chromosome 2, chromosome 5, chromosome 4 and chromosome 6, respectively.

Candidate genes were mapped to the reference genome and most annotated genes were located on other five chromosomes except the chromosome 3 (**Supplementary Figure S1**; **Supplementary Table 11**). Twenty-five genes were located on chromosome 1L (the long arm of chromosome 1) and 17 genes were located on chromosome 1S (the short arm of chromosome 1). Seven, eleven, one and seven of these annotated genes were located on chromosome 2, chromosome 4, chromosome 5, chromosome 6, respectively. Furthermore, there were also seven genes located on free chromosomes (the unassigned scaffolds that cannot be placed on any known chromosome).

## 5 Discussion

### 5.1 The first ultra-dense genetic map for faba bean

Owing to the rapid development of high-throughput sequencing technologies, sufficient molecular markers can now be obtained to facilitate the mapping of high-density genetic maps and research on map-based gene cloning (Yang et al., 2012; Zhang et al., 2016; Zhou et al., 2018; Gaur et al., 2020; Gu et al., 2020; Sa et al., 2021). The molecular genetic analysis of faba bean is currently lagging in comparison to that of many other crops due to its large genome size (Adhikari et al., 2021). Establishing a reliable linkage map between genetic markers and traits is one of the key approaches to improve molecular breeding without a reference genome (Chapman et al., 2022). In this study, a genetic map of faba beans was constructed using high-throughput genotyping platforms. To date, genetic map construction using microarray chips has been successfully reported in several crops, such as pea (Tayeh et al., 2015), wheat (Liu et al., 2018; Ren et al., 2021), cotton (Gu et al., 2020) and pepper (Cheng et al., 2016). In addition, the 130 K liquid-phase gene chip used in this study was developed using transcriptome data, which contains large-scale information. Furthermore, all marker sequences provided valuable gene information, indicating that this liquid-phase gene chip is an effective and feasible tool to utilize for genetic map construction.

There have been more than 20 genetic maps reported for faba beans. Of these, the genetic map constructed by Carrillo-perdomo et al. (2020) containing 1,728 markers, with a total length of 1,547.71 cM and an average genetic distance of 0.89 cM. To date, one of the two SNP genetic map constructed by Li et al. (2023) had the highest density, containing 5,103 markers, with a total length of 1,333.31 cM and an average genetic distance of 0.26 cM. In the present study, an ultra-dense genetic map was constructed, encompassing 12,023 markers in 6 LGs, with an average distance of 0.098 cM. The number, density, and distribution quality of the new molecular markers was thus significantly higher when compared with previous genetic maps. The presented genetic map only has 9 gaps > 5 cM, and thus, it can be effectively utilized for faba bean gene mapping and MAS breeding.

### 5.2 Comparison with previous QTL reports

QTL mapping and the analysis of candidate genes within QTL intervals is an effective strategy to investigate numerous crop traits (Bornowski et al., 2020; Chen et al., 2021), and can contribute to the development of molecular marker-assisted breeding (Torres et al., 2010). The 100-seed weight, seed shape, and nutritional quality of faba beans are all quantitative traits susceptible to environmental influence. To improve the accuracy of QTL mapping for seed traits, QTL analysis was performed in the F_2_ and F_2:3_ populations in two locations. There was a total of 65 seed trait-related QTLs detected (**Supplementary Table S6**), of which, 11 were repeatedly detected in different environments (**Supplementary Table S9**).

Patto et al. (1999) used a genetic map constructed using the F_2_ population and found that most of the QTLs related to seed weight were located on chromosome 6 for faba bean. Using the recombinant in bred line (RIL6) population constructed using Vf6 and Vf27, Ávila et al. (2017) identified 5 QTLs for HSW, which were located on 4 different chromosomes. Tian et al. (2018) identified two QTLs for seed weight using an F_2_ population derived from Yun122/TF42, which were located on two different LGs. In this study, we identified three QTLs linked to HSW, one at LG4, and two at LG5. These results indicate that faba bean seed weight is controlled by multiple main-effect QTLs. *qHSW5.1*, one of the three QTLs related to HSW, was also associated with SA, and *qHSW5.2* was associated with SA, SP, SL, and SW, which indicated that these two QTLs are also involved in controlling seed shape (**Table 3**).

Seed shape traits are among the most important factors used to determine seed size. The localization and cloning of seed shape genes are of great importance when aiming to increase crop yield and improve appearance quality (Austin and Lee, 1996; Song et al., 2007; Verma et al., 2015; Cheng et al., 2017; Murube et al., 2020). According to the Gramene website (http://archive.gramene.org/qtl/), more than 400 rice grain shape-related genes/QTLs have been identified through genetic mapping and correlation analysis. However, few studies have reported QTL mapping for the seed shape traits of faba bean, a seed length-related QTL and a seed width-related QTL were identified by Tian et al. (2018), 8 QTLs related to seed length, 9 QTLs related to seed width and 8 QTLs related to seed thickness were identified by Li et al. (2023). In this investigation, 28 QTLs for 6 seed-shape traits were identified using linkage analysis, and most were located on LG5(**Table 3**; **Supplementary Table S6**). Compared to these QTLs reported, those identified as controlling seed shape in this study were new, and could thus be applied to the subsequent fine mapping of seed shape traits and the investigation of related genes in faba bean. *qSA5.1*, *qSLWR6.1* and *qST5.1* were stable QTLs explained > 10% of phenotypic variation, while *qSA5.1* was also associated with SP, SL, and SW. which indicated that these QTLs can be used for further fine mapping and superior gene discovery of seed shape traits.

Seed coat color is a key factor affecting seed quality (Yoshimura et al., 2012; García-Fernández et al., 2021). Different seed coat colors may have different functions (Debeaujon et al., 2003), and the different seed coat colors of faba beans may also be associated with different nutritional qualities. The results of the correlation analysis among seed traits showed that seed coat color was positively correlated with FC and StC, and negatively correlated with LC. Mendel first proposed that the seed coat color of peas is controlled by a pair of genes and considered a qualitative trait (Myers, 2004), while the seed coat color of soybeans is controlled by multiple genetic loci (Choung et al., 2001), and more than 30 molecular marker loci on different chromosomes that control seed coat color in soybean have been detected (Yuan et al., 2022). However, few studies on the QTLs for seed coat color in faba bean have been reported. WY7 and TCX7, the parents used in this study, have purple and white coats, respectively. A total of 12 QTLs, mainly located on LG1, were detected by quantitative measurement of the SC-R, SC-G, and SC-B. *qSC-R1.2* was also located with SC-G and SC-B (**Table 3**), which could explain the > 50% phenotypic variation. *qSC-R1.1* is located with SC-G, and *qSC-R1.3* is located with SC-B (**Supplementary Table S6**). These three QTLs are key objects for further study of grain coat color traits.

The main nutrients in faba bean seeds are protein and starch, with low lipid and fiber content levels, as well as tannin (Zanotto et al., 2020), pyrimidine glucoside, and other bioactive substances (Björnsdotter et al., 2021). QTL mapping for quality traits can help to improve the utilization and value of faba beans. At present, there are relatively few studies on the QTL mapping of quality traits in broad beans. Only five genes that control grain proteins have been identified (Macas et al. 1993b). In this study, 22 QTLs linked to quality traits were detected using SNP markers for the first time, including 7 QTLs for FC, 7 for StC, 4 for LC, 2 for PC, and 2 for TC (**Supplementary Table S6**). In particular, *qFC1.1*, *qTC1.1*, *qLC1.1*, and *qLC3.1* could explain > 20% of the phenotypic variation, and *qStC1.1* was also associated with PC (**Table 3**). These QTLs could thus be used to identify the candidate genes for faba bean quality traits.

### 5.3 Candidate genes for the QTLs controlling seed-related traits

To identify candidate genes for seed-related traits in faba bean, we focused on 213 genes within 41 QTL intervals that explained > 10% of the phenotypic variation. According to the results of the functional annotation, 57.28% of these genes had been annotated. Signaling pathways that regulate seed size in plants include the ubiquitin-protease pathway, mitogen-activated protein kinase signaling pathway, transcriptional regulation, G-protein signaling pathway, IKU pathway, and plant hormones (Gnan et al., 2014; Li and Li, 2016; Li et al., 2019). Jayakodi et al. (2023) identified 15 marker–seed size associations, and most prominent signal was located on chromosome 4 within the *Vfaba.Hedin2.R1.4g051440* gene. In this investigation, there were 30 genes annotations among the 59 genes linked to HSW and seed shape (**Supplementary Table S11**). Thirteen of these annotation genes located on chromosome 4 by whole genome sequence alignment. dou_TRINITY_DN52935_c3_g4 and hua_TRINITY_DN119282_c0_g1 encode serine/threonine phosphatase and the transcription factor bHLH, respectively, which are reportedly involved in regulating seed size (Savadi, 2018). dou_TRINITY_DN38848_c0_g1 encodes a *CYP* gene and *CYP* is involved in protein folding, signal transduction, and RNA processing (Krücken et al., 2009). There are also two calcium signaling pathway genes, including a calcium-binding protein gene ye_TRINITY_DN120969_c0_g1 and a Ca^2+^/H^+^-exchanging protein gene hua_TRINITY_DN154 119_c0_g2, which may be involved in the Ca signaling pathway to regulate seed development. Other unannotated candidate genes could also potentially regulate seed size.

The seed coat color of plants is affected by numerous factors, but flavonoids are the decisive pigments (Lepiniec et al., 2006). In this study, there were 34 candidate genes for seed coat color, 19 of which were annotated (**Supplementary Table S11**). Among these genes, the translated product of ye_TRINITY_DN150431_c0_g1 is a ubiquitin-like protein that plays an important role in pigment accumulation (Tang et al., 2015). ye_TRINITY_DN150347_c0_g1 and ye_TRINITY_DN139828_c0_g1 are WD40 family genes, which have been suggested to regulate the formation of proanthocyanidins in seed coats (Shirley et al., 1995; Walker et al., 1999). Furthermore, the other 16 annotated genes and 15 unannotated genes may also be required for the pigment composition of different seed coat colors, but this requires further verification.

In this study, 79 candidate genes were associated with five nutritional quality traits, of which, 41 were annotated (**Supplementary Table S11**). There were 7 genes for LC, 4 of which were annotated, but no functions related to lipid synthesis and accumulation were reported. hua_TRINITY_DN145176_c0_g1, a crude fiber candidate gene, is a triose-phosphate transporter gene that reportedly affects starch and glucose transport in transgenic tobacco (Häusler et al., 1998). dou_TRINITY_DN53089_c3_g1 and ye_T RINITY_DN155843_c1_g1 are GDSL esterases that may also be involved in fiber metabolism. Condensed tannins, also known as proanthocyanidins, exhibit antioxidant, antibacterial, anticancer, and anti-mutation activities (Gutierrez et al., 2020). The two genes zt-1 and zt-2 are the most studied for controlling tannin content in faba bean (Gutierrez et al., 2006; Gutierrez et al., 2007; Gutierrez et al., 2008). Of the candidate genes related to tannins, dou_TRINITY_DN58315_c1_g1 encodes a bHLH transcription factor gene, which is reportedly involved in the mechanisms of tannin biosynthesis in faba bean (Gutierrez et al., 2020). Other tannin-annotated genes obtained in the target intervals have not been reported in faba bean, and thus may be candidate genes affecting tannin content. Further studies are required to confirm the functions of these genes.

### 5.4 Reference genome mapping analysis

Compared to chromosomes and gene locations of the reference genome, the number of linkage groups in our genetic map was consistent with their respective chromosomes, but there were variations in the order of genes on the chromosome, and about 25% of them were not found in the genome (**Supplementary Table S12**). Eighty-five candidate genes within the QTL interval were mapped to the reference genome, seven of which were located on the contigs (**Supplementary Figure S1**). Therefore, a part of contigs on the reference genome can be assembled to the genome of faba bean based on the map constructed in this study, which is conducive to the further improvement of the physical map of faba bean.

## 6 Conclusions

A high-density genetic map with 12,023 SNPs in 6 LGs was constructed using the faba_bean_ 130 K SNP TNGS genotyping platform. A total of 65 QTLs for seed-related traits were identified (3 for 100-seed weight, 28 for seed shape, 12 for seed coat color, and 22 for nutritional quality). Furthermore, 333 candidate genes were identified that are likely to participate in the regulation of seed-related traits. This is the first ultra-dense genetic map of faba bean and it provides a foundation for further genetic analyses, MAS breeding, and reference genome assembly research. This study will also be useful for faba bean gene isolation and functional genomics research.

## Data availability statement

The datasets presented in this study can be found in online repositories. The names of the names of the repository/repositories and accession number(s) can be found in the article/**Supplementary Material**.

## Author contributions

NZ and LW conceived and designed the experiments. DX, YM, YW, YZ, MY and CG performed experiment, EZ, KW and BL analysed data. NZ wrote the manuscript. LW and XW revised the manuscript. All authors contributed to the article and approved the submitted version.
