# Supplementary figures and images for: Construction of a high-density genetic map for faba bean (Vicia faba L.) and quantitative trait loci mapping of seed-related traits
Source: Front Plant Sci. 2023 Jun 7;14:1201103. doi: 10.3389/fpls.2023.1201103 (PMC10282779; doi:10.3389/fpls.2023.1201103)

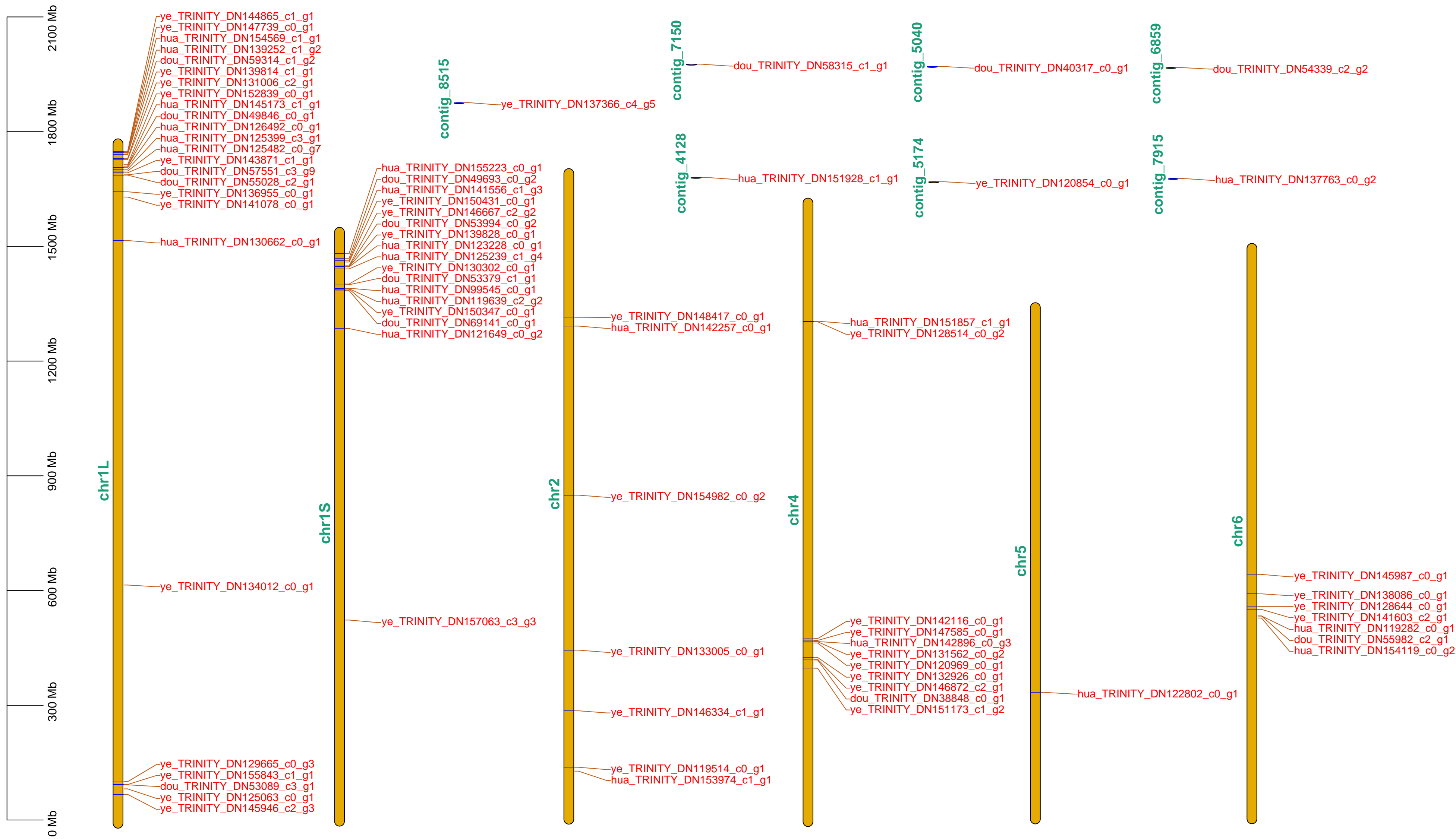

Supplement: Supplementary file 1 [file DataSheet_1.pdf]
